# (*R*)-1,1′-Binaphthalene-2,2′-diol–(*Z*)-*N*-ethyl­ideneethanamine *N*-oxide (1/1)

**DOI:** 10.1107/S1600536812007477

**Published:** 2012-03-03

**Authors:** Bo Shao, Hai-Bin Wang

**Affiliations:** a College of Biology and Environmental Engineering, Zhejiang Shuren University, Hangzhou 310015, People’s Republic of China; b College of Chemical Engineering and Materials Science, Zhejiang University of Technology, Hangzhou 310014, People’s Republic of China

## Abstract

In the title compound, C_4_H_9_NO·C_20_H_14_O_2_, the dihedral angle between the naphthalene ring systems of the binaphthalene­diol mol­ecule is 77.53 (14)°. In the crystal, the two components are linked by O—H⋯O hydrogen bonds, forming a zigzag chain along the *c* axis.

## Related literature

For applications of 2,2′-dihy­droxy-1,1′-dinaphthalene in asymmetric synthesis, see: Noyori *et al.* (1984 ▶); Reeder *et al.* (1994 ▶); Toda *et al.* (1989 ▶); Zhang & Schuster (1994 ▶). For related literature on oxidation of hydroxyl­amines to nitro­nes, see: Cicchi *et al.* (2001 ▶); Engel *et al.* (1997 ▶); Liu *et al.* (2004 ▶).
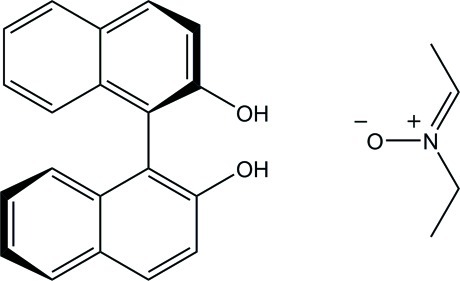


## Experimental

### 

#### Crystal data

C_4_H_9_NO·C_20_H_14_O_2_
*M* *_r_* = 373.43
Trigonal, 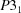

*a* = 8.9579 (6) Å
*c* = 21.187 (3) Å
*V* = 1472.4 (2) Å^3^
*Z* = 3
Mo *K*α radiation
μ = 0.08 mm^−1^
*T* = 273 K
0.31 × 0.22 × 0.18 mm

#### Data collection

Bruker APEX area-detector diffractometer
Absorption correction: multi-scan (*SADABS*; Bruker, 2001 ▶) *T*_min_ = 0.964, *T*_max_ = 0.977
7771 measured reflections
1732 independent reflections
1662 reflections with *I* > 2σ(*I*)
*R*_int_ = 0.033

#### Refinement

*R*[*F* ^2^ > 2σ(*F* ^2^)] = 0.053
*wR*(*F* ^2^) = 0.115
*S* = 1.19
1732 reflections
257 parameters
1 restraint
H-atom parameters constrained
Δρ_max_ = 0.19 e Å^−3^
Δρ_min_ = −0.26 e Å^−3^

### 

Data collection: *SMART* (Bruker, 2007 ▶); cell refinement: *SAINT* (Bruker, 2007 ▶); data reduction: *SAINT*; program(s) used to solve structure: *SHELXS97* (Sheldrick, 2008 ▶); program(s) used to refine structure: *SHELXL97* (Sheldrick, 2008 ▶); molecular graphics: *SHELXTL* (Sheldrick, 2008 ▶); software used to prepare material for publication: *SHELXL97*.

## Supplementary Material

Crystal structure: contains datablock(s) global, I. DOI: 10.1107/S1600536812007477/is5033sup1.cif

Structure factors: contains datablock(s) I. DOI: 10.1107/S1600536812007477/is5033Isup2.hkl

Additional supplementary materials:  crystallographic information; 3D view; checkCIF report

## Figures and Tables

**Table 1:** Hydrogen-bond geometry (Å, °)

*D*—H⋯*A*	*D*—H	H⋯*A*	*D*⋯*A*	*D*—H⋯*A*
O1—H1⋯O3	0.82	1.90	2.706 (5)	167
O2—H2⋯O3^i^	0.82	1.96	2.763 (5)	168

Symmetry code: (i) 
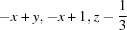
.

**Fig. 1.**
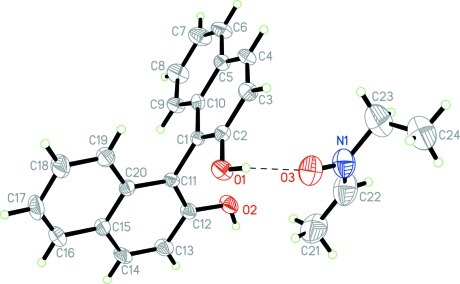
The molecular structure of the title compound, with atom-labeling and displacement ellipsoids drawn at the 40% probability level. The hydrogen bond is illustrated as a dashed line.

**Fig. 2.**
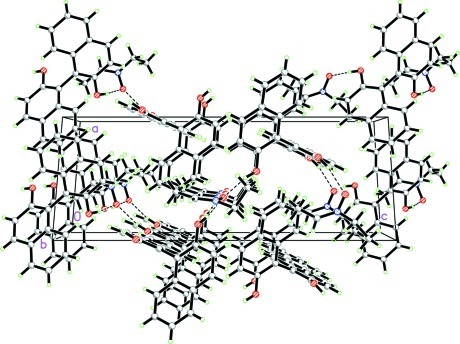
A crystal packing diagram of the title compound, viewed along the b axis. Hydrogen bonds are drawn as dashed lines.

## supplementary crystallographic information

### Comment

2,2'-Dihydroxy-1,1'-dinaphthalene (binaphthol) is an important chemical as a precursor for catalysis in asymmetric synthesis, as a chiral host for molecular recognition and enantiomer separation and also as intermediates for the synthesis of chiral materials (Noyori *et al.*, 1984; Toda *et al.*, 1989; Reeder *et al.*, 1994; Zhang & Schuster, 1994). *N*,*N*-diethylhydroxylamine is easily oxidized to form *E* and *Z* type of *N*-ethylideneethanamine N-oxide (ELDEA) (Engel *et al.*, 1997; Liu *et al.*, 2004; Cicchi *et al.*, 2001). In this study, only the *Z* type of ELDEA is trapped by the (*R*)-binaphthol to form the title complex, (I) (Scheme 1 and Fig. 1).

The asymmetric unit comprises a ELDEA (*Z* type) molecule and a (*R*)-binaphthol molecule, which are linked by an intermolecular O—H···O hydrogen bond (Table 1). The two naphthyl groups are almost perpendicular to each other with a dihedral angle being 77.53 (14)°. The N1—O3 length [1.313 (5) Å] of ELDEA is characteristic of the N—O single bond, while the N1═C22 bond [1.265 (7) Å] is a double bond. Because of the existence of N1═C22 double bond, the O3/N1/C21/C22/C24 plane is almost planar. The ELDEA is connected to two H atoms from different binaphthol molecules through O—H···O hydrogen bonds (Fig. 2 and Table 1), forming infinite chains running along the *c* axis.

### Experimental

The (*R*)-1,1'-bi-2-naphthol (2.9 g) and *N*,*N*-diethylhydroxylamine (0.9 g) were mixed and dissolved in sufficient ethanol 30 ml by heating to a temperature of 353 K where a clear solution was resulted, then refluxed for 5 h. The materials of experiment were exposed in air. Single crystals of (I) (2.7 g) were formed form an ethanol solution by gradual evaporation for two weeks at room temperature.

### Refinement

All H atoms were placed in calculated positions and allowed to ride on their parent atoms, with O—H = 0.82 Å, and C—H = 0.93 Å (aromatic), 0.96 Å (methyl) and 0.97 Å (methylene), and with *U*_iso_(H) = 1.2 or 1.5*U*_eq_ of the parent atom. In the absence of significant anomalous scattering, Friedel pairs were merged before the final refinement.

### Crystal data

**Table:**

C_4_H_9_NO·C_20_H_14_O_2_	*D*_x_ = 1.263 Mg m^−^^3^
*M**_r_* = 373.43	Mo *K*α radiation, λ = 0.71073 Å
Trigonal, *P*3_1_	Cell parameters from 1360 reflections
Hall symbol: P 31	θ = 2.4–16.6°
*a* = 8.9579 (6) Å	µ = 0.08 mm^−^^1^
*c* = 21.187 (3) Å	*T* = 273 K
*V* = 1472.4 (2) Å^3^	Prism, colorless
*Z* = 3	0.31 × 0.22 × 0.18 mm
*F*(000) = 594.0	

### Data collection

**Table:**

Bruker APEX area-detector diffractometer	1732 independent reflections
Radiation source: fine-focus sealed tube	1662 reflections with *I* > 2σ(*I*)
Graphite monochromator	*R*_int_ = 0.033
φ and ω scan	θ_max_ = 25.0°, θ_min_ = 2.6°
Absorption correction: multi-scan (*SADABS*; Bruker, 2001)	*h* = −10→9
*T*_min_ = 0.964, *T*_max_ = 0.977	*k* = −7→10
7771 measured reflections	*l* = −25→25

### Refinement

**Table:**

Refinement on *F*^2^	Primary atom site location: structure-invariant direct methods
Least-squares matrix: full	Secondary atom site location: difference Fourier map
*R*[*F*^2^ > 2σ(*F*^2^)] = 0.053	Hydrogen site location: inferred from neighbouring sites
*wR*(*F*^2^) = 0.115	H-atom parameters constrained
*S* = 1.19	*w* = 1/[σ^2^(*F*_o_^2^) + (0.0471*P*)^2^ + 0.4671*P*] where *P* = (*F*_o_^2^ + 2*F*_c_^2^)/3
1732 reflections	(Δ/σ)_max_ < 0.001
257 parameters	Δρ_max_ = 0.19 e Å^−^^3^
1 restraint	Δρ_min_ = −0.26 e Å^−^^3^

### Special details

**Table:**

Experimental. The IR (KBr pellet) spectrum of (I) showed bands: 3069, 1624, 1583, 1504, 1433, 1338, 1275, 1241, 1177, 1142, 1093, 1012, 979, 965, 928, 867, 820, 753, 624, 581, 492, 442 and 424 cm^-1^.
Geometry. All e.s.d.'s (except the e.s.d. in the dihedral angle between two l.s. planes) are estimated using the full covariance matrix. The cell e.s.d.'s are taken into account individually in the estimation of e.s.d.'s in distances, angles and torsion angles; correlations between e.s.d.'s in cell parameters are only used when they are defined by crystal symmetry. An approximate (isotropic) treatment of cell e.s.d.'s is used for estimating e.s.d.'s involving l.s. planes.
Refinement. Refinement of *F*^2^ against ALL reflections. The weighted *R*-factor *wR* and goodness of fit *S* are based on *F*^2^, conventional *R*-factors *R* are based on *F*, with *F* set to zero for negative *F*^2^. The threshold expression of *F*^2^ > σ(*F*^2^) is used only for calculating *R*-factors(gt) *etc*. and is not relevant to the choice of reflections for refinement. *R*-factors based on *F*^2^ are statistically about twice as large as those based on *F*, and *R*- factors based on ALL data will be even larger.

### Fractional atomic coordinates and isotropic or equivalent isotropic displacement parameters (Å^2^)

**Table:**

	*x*	*y*	*z*	*U*_iso_*/*U*_eq_	
O1	0.6567 (4)	0.7994 (4)	0.76094 (13)	0.0418 (7)	
H1	0.5835	0.7851	0.7872	0.063*	
O2	0.5514 (4)	0.5899 (4)	0.59216 (14)	0.0500 (8)	
H2	0.5018	0.5977	0.5609	0.075*	
O3	0.3797 (4)	0.7314 (5)	0.83262 (16)	0.0582 (9)	
N1	0.2490 (6)	0.5834 (6)	0.8144 (2)	0.0643 (12)	
C1	0.7392 (4)	0.6275 (4)	0.70313 (16)	0.0255 (7)	
C2	0.6670 (5)	0.6537 (5)	0.75628 (16)	0.0308 (8)	
C3	0.6065 (5)	0.5327 (5)	0.80628 (17)	0.0365 (9)	
H3A	0.5573	0.5521	0.8417	0.044*	
C4	0.6198 (5)	0.3880 (5)	0.80293 (17)	0.0356 (9)	
H4	0.5799	0.3100	0.8363	0.043*	
C5	0.6929 (5)	0.3548 (5)	0.74984 (19)	0.0344 (9)	
C6	0.7057 (6)	0.2039 (6)	0.7449 (2)	0.0472 (11)	
H6	0.6660	0.1247	0.7778	0.057*	
C7	0.7742 (7)	0.1731 (6)	0.6935 (2)	0.0551 (12)	
H7	0.7819	0.0735	0.6911	0.066*	
C8	0.8336 (7)	0.2911 (6)	0.6437 (2)	0.0510 (12)	
H8	0.8796	0.2686	0.6080	0.061*	
C9	0.8253 (5)	0.4385 (5)	0.64668 (18)	0.0370 (9)	
H9	0.8674	0.5161	0.6132	0.044*	
C10	0.7537 (4)	0.4758 (5)	0.69961 (17)	0.0292 (8)	
C11	0.8048 (5)	0.7566 (4)	0.65051 (16)	0.0266 (8)	
C12	0.7106 (5)	0.7323 (5)	0.59651 (17)	0.0323 (9)	
C13	0.7759 (5)	0.8506 (5)	0.54687 (18)	0.0411 (10)	
H13	0.7108	0.8299	0.5104	0.049*	
C14	0.9330 (5)	0.9954 (5)	0.55105 (18)	0.0399 (10)	
H14	0.9747	1.0715	0.5172	0.048*	
C15	1.0329 (5)	1.0315 (5)	0.60593 (19)	0.0342 (9)	
C16	1.1919 (6)	1.1874 (6)	0.6136 (2)	0.0464 (11)	
H16	1.2330	1.2674	0.5810	0.056*	
C17	1.2848 (6)	1.2220 (6)	0.6673 (2)	0.0562 (13)	
H17	1.3878	1.3256	0.6718	0.067*	
C18	1.2245 (6)	1.1009 (6)	0.7159 (2)	0.0579 (13)	
H18	1.2897	1.1232	0.7525	0.069*	
C19	1.0714 (5)	0.9500 (6)	0.71080 (19)	0.0413 (10)	
H19	1.0337	0.8720	0.7441	0.050*	
C20	0.9695 (5)	0.9104 (5)	0.65601 (17)	0.0288 (8)	
C21	0.2757 (9)	0.6739 (10)	0.7052 (3)	0.094 (2)	
H21A	0.3380	0.6372	0.6785	0.140*	
H21B	0.3534	0.7868	0.7215	0.140*	
H21C	0.1865	0.6772	0.6812	0.140*	
C22	0.1986 (8)	0.5529 (9)	0.7577 (3)	0.0802 (18)	
H22	0.1050	0.4449	0.7486	0.096*	
C23	0.0453 (10)	0.4797 (11)	0.9012 (4)	0.110 (3)	
H23A	−0.0384	0.4820	0.8735	0.165*	
H23B	0.1082	0.5886	0.9225	0.165*	
H23C	−0.0123	0.3896	0.9318	0.165*	
C24	0.1635 (9)	0.4481 (9)	0.8648 (3)	0.093 (2)	
H24A	0.1012	0.3351	0.8454	0.112*	
H24B	0.2505	0.4498	0.8925	0.112*	

### Atomic displacement parameters (Å^2^)

**Table:**

	*U*^11^	*U*^22^	*U*^33^	*U*^12^	*U*^13^	*U*^23^
O1	0.0524 (18)	0.0364 (15)	0.0433 (17)	0.0272 (14)	0.0132 (13)	0.0028 (12)
O2	0.0400 (16)	0.0387 (17)	0.0457 (18)	0.0005 (13)	−0.0158 (13)	0.0099 (13)
O3	0.057 (2)	0.067 (2)	0.055 (2)	0.0340 (19)	0.0085 (16)	−0.0168 (16)
N1	0.055 (3)	0.072 (3)	0.057 (3)	0.025 (2)	0.007 (2)	−0.006 (2)
C1	0.0221 (17)	0.0219 (18)	0.0266 (17)	0.0067 (15)	−0.0059 (15)	−0.0002 (14)
C2	0.0277 (19)	0.032 (2)	0.033 (2)	0.0144 (17)	−0.0027 (15)	0.0006 (15)
C3	0.042 (2)	0.045 (2)	0.0238 (19)	0.0221 (19)	0.0043 (16)	0.0013 (16)
C4	0.041 (2)	0.035 (2)	0.028 (2)	0.0174 (18)	0.0057 (17)	0.0098 (17)
C5	0.032 (2)	0.030 (2)	0.038 (2)	0.0134 (17)	−0.0003 (17)	0.0037 (16)
C6	0.061 (3)	0.035 (2)	0.048 (3)	0.026 (2)	0.006 (2)	0.0130 (19)
C7	0.078 (3)	0.042 (3)	0.058 (3)	0.040 (3)	0.011 (3)	0.006 (2)
C8	0.071 (3)	0.048 (3)	0.045 (3)	0.038 (3)	0.011 (2)	−0.002 (2)
C9	0.045 (2)	0.036 (2)	0.033 (2)	0.0229 (19)	0.0051 (17)	0.0049 (17)
C10	0.0246 (18)	0.0260 (19)	0.032 (2)	0.0093 (15)	−0.0003 (15)	0.0000 (15)
C11	0.0305 (19)	0.0234 (18)	0.0277 (18)	0.0148 (16)	0.0031 (15)	0.0026 (14)
C12	0.032 (2)	0.028 (2)	0.032 (2)	0.0116 (17)	−0.0026 (16)	−0.0003 (16)
C13	0.045 (2)	0.043 (2)	0.030 (2)	0.018 (2)	−0.0035 (18)	0.0063 (18)
C14	0.045 (2)	0.040 (2)	0.029 (2)	0.017 (2)	0.0055 (18)	0.0156 (18)
C15	0.031 (2)	0.029 (2)	0.040 (2)	0.0129 (17)	0.0030 (17)	0.0030 (16)
C16	0.039 (2)	0.037 (2)	0.052 (3)	0.0106 (19)	0.008 (2)	0.0136 (19)
C17	0.033 (2)	0.040 (2)	0.067 (3)	−0.003 (2)	−0.007 (2)	0.003 (2)
C18	0.041 (3)	0.049 (3)	0.058 (3)	0.003 (2)	−0.016 (2)	0.005 (2)
C19	0.039 (2)	0.041 (2)	0.035 (2)	0.0126 (19)	−0.0064 (17)	0.0014 (17)
C20	0.0285 (19)	0.0288 (19)	0.032 (2)	0.0168 (16)	0.0016 (16)	−0.0009 (15)
C21	0.076 (4)	0.149 (6)	0.058 (3)	0.057 (5)	−0.007 (3)	0.016 (4)
C22	0.055 (3)	0.097 (5)	0.070 (4)	0.025 (3)	−0.012 (3)	−0.021 (4)
C23	0.095 (5)	0.110 (6)	0.119 (6)	0.047 (5)	0.032 (5)	0.033 (5)
C24	0.092 (5)	0.087 (5)	0.088 (5)	0.035 (4)	0.024 (4)	0.014 (4)

### Geometric parameters (Å, º)

**Table:**

O1—C2	1.357 (4)	C12—C13	1.397 (5)
O1—H1	0.8200	C13—C14	1.358 (6)
O2—C12	1.360 (5)	C13—H13	0.9300
O2—H2	0.8200	C14—C15	1.403 (6)
O3—N1	1.313 (5)	C14—H14	0.9300
N1—C22	1.265 (7)	C15—C20	1.417 (5)
N1—C24	1.506 (8)	C15—C16	1.420 (6)
C1—C2	1.376 (5)	C16—C17	1.351 (6)
C1—C10	1.431 (5)	C16—H16	0.9300
C1—C11	1.498 (5)	C17—C18	1.394 (7)
C2—C3	1.416 (5)	C17—H17	0.9300
C3—C4	1.361 (6)	C18—C19	1.366 (6)
C3—H3A	0.9300	C18—H18	0.9300
C4—C5	1.406 (6)	C19—C20	1.408 (5)
C4—H4	0.9300	C19—H19	0.9300
C5—C6	1.416 (6)	C21—C22	1.463 (9)
C5—C10	1.419 (5)	C21—H21A	0.9600
C6—C7	1.345 (6)	C21—H21B	0.9600
C6—H6	0.9300	C21—H21C	0.9600
C7—C8	1.397 (7)	C22—H22	0.9300
C7—H7	0.9300	C23—C24	1.447 (10)
C8—C9	1.361 (6)	C23—H23A	0.9600
C8—H8	0.9300	C23—H23B	0.9600
C9—C10	1.412 (5)	C23—H23C	0.9600
C9—H9	0.9300	C24—H24A	0.9700
C11—C12	1.373 (5)	C24—H24B	0.9700
C11—C20	1.434 (5)		
			
C2—O1—H1	109.5	C12—C13—H13	119.4
C12—O2—H2	109.5	C13—C14—C15	120.7 (3)
C22—N1—O3	122.6 (5)	C13—C14—H14	119.6
C22—N1—C24	121.1 (5)	C15—C14—H14	119.6
O3—N1—C24	116.3 (4)	C14—C15—C20	118.6 (3)
C2—C1—C10	118.6 (3)	C14—C15—C16	122.2 (4)
C2—C1—C11	120.9 (3)	C20—C15—C16	119.2 (4)
C10—C1—C11	120.5 (3)	C17—C16—C15	121.5 (4)
O1—C2—C1	119.2 (3)	C17—C16—H16	119.2
O1—C2—C3	119.9 (3)	C15—C16—H16	119.2
C1—C2—C3	120.9 (3)	C16—C17—C18	119.3 (4)
C4—C3—C2	120.5 (3)	C16—C17—H17	120.4
C4—C3—H3A	119.7	C18—C17—H17	120.4
C2—C3—H3A	119.7	C19—C18—C17	121.2 (4)
C3—C4—C5	121.0 (3)	C19—C18—H18	119.4
C3—C4—H4	119.5	C17—C18—H18	119.4
C5—C4—H4	119.5	C18—C19—C20	121.2 (4)
C4—C5—C6	122.0 (4)	C18—C19—H19	119.4
C4—C5—C10	118.7 (3)	C20—C19—H19	119.4
C6—C5—C10	119.3 (4)	C19—C20—C15	117.6 (4)
C7—C6—C5	121.3 (4)	C19—C20—C11	122.3 (3)
C7—C6—H6	119.4	C15—C20—C11	120.1 (3)
C5—C6—H6	119.4	C22—C21—H21A	109.5
C6—C7—C8	119.8 (4)	C22—C21—H21B	109.5
C6—C7—H7	120.1	H21A—C21—H21B	109.5
C8—C7—H7	120.1	C22—C21—H21C	109.5
C9—C8—C7	120.9 (4)	H21A—C21—H21C	109.5
C9—C8—H8	119.5	H21B—C21—H21C	109.5
C7—C8—H8	119.5	N1—C22—C21	125.2 (6)
C8—C9—C10	121.2 (4)	N1—C22—H22	117.4
C8—C9—H9	119.4	C21—C22—H22	117.4
C10—C9—H9	119.4	C24—C23—H23A	109.5
C9—C10—C5	117.4 (3)	C24—C23—H23B	109.5
C9—C10—C1	122.3 (3)	H23A—C23—H23B	109.5
C5—C10—C1	120.3 (3)	C24—C23—H23C	109.5
C12—C11—C20	118.4 (3)	H23A—C23—H23C	109.5
C12—C11—C1	121.7 (3)	H23B—C23—H23C	109.5
C20—C11—C1	120.0 (3)	C23—C24—N1	110.4 (6)
O2—C12—C11	118.6 (3)	C23—C24—H24A	109.6
O2—C12—C13	120.4 (3)	N1—C24—H24A	109.6
C11—C12—C13	121.1 (3)	C23—C24—H24B	109.6
C14—C13—C12	121.1 (4)	N1—C24—H24B	109.6
C14—C13—H13	119.4	H24A—C24—H24B	108.1
			
C10—C1—C2—O1	178.3 (3)	C20—C11—C12—O2	−177.4 (3)
C11—C1—C2—O1	−0.5 (5)	C1—C11—C12—O2	2.2 (5)
C10—C1—C2—C3	−0.6 (5)	C20—C11—C12—C13	2.7 (5)
C11—C1—C2—C3	−179.4 (3)	C1—C11—C12—C13	−177.7 (3)
O1—C2—C3—C4	−178.4 (4)	O2—C12—C13—C14	178.5 (4)
C1—C2—C3—C4	0.5 (6)	C11—C12—C13—C14	−1.6 (6)
C2—C3—C4—C5	−0.3 (6)	C12—C13—C14—C15	−1.0 (6)
C3—C4—C5—C6	−179.0 (4)	C13—C14—C15—C20	2.4 (6)
C3—C4—C5—C10	0.3 (6)	C13—C14—C15—C16	−175.4 (4)
C4—C5—C6—C7	179.2 (5)	C14—C15—C16—C17	178.4 (5)
C10—C5—C6—C7	−0.1 (7)	C20—C15—C16—C17	0.7 (7)
C5—C6—C7—C8	−0.2 (8)	C15—C16—C17—C18	1.0 (8)
C6—C7—C8—C9	0.8 (8)	C16—C17—C18—C19	−1.7 (8)
C7—C8—C9—C10	−1.1 (7)	C17—C18—C19—C20	0.6 (8)
C8—C9—C10—C5	0.8 (6)	C18—C19—C20—C15	1.1 (6)
C8—C9—C10—C1	−178.3 (4)	C18—C19—C20—C11	−177.2 (4)
C4—C5—C10—C9	−179.5 (4)	C14—C15—C20—C19	−179.5 (4)
C6—C5—C10—C9	−0.2 (5)	C16—C15—C20—C19	−1.7 (5)
C4—C5—C10—C1	−0.5 (5)	C14—C15—C20—C11	−1.2 (5)
C6—C5—C10—C1	178.9 (4)	C16—C15—C20—C11	176.6 (4)
C2—C1—C10—C9	179.7 (3)	C12—C11—C20—C19	177.0 (4)
C11—C1—C10—C9	−1.6 (5)	C1—C11—C20—C19	−2.7 (5)
C2—C1—C10—C5	0.6 (5)	C12—C11—C20—C15	−1.2 (5)
C11—C1—C10—C5	179.4 (3)	C1—C11—C20—C15	179.1 (3)
C2—C1—C11—C12	−100.6 (4)	O3—N1—C22—C21	0.1 (10)
C10—C1—C11—C12	80.6 (4)	C24—N1—C22—C21	−178.4 (6)
C2—C1—C11—C20	79.0 (4)	C22—N1—C24—C23	−100.9 (8)
C10—C1—C11—C20	−99.7 (4)	O3—N1—C24—C23	80.5 (7)

### Hydrogen-bond geometry (Å, º)

**Table:**

*D*—H···*A*	*D*—H	H···*A*	*D*···*A*	*D*—H···*A*
O1—H1···O3	0.82	1.90	2.706 (5)	167
O2—H2···O3^i^	0.82	1.96	2.763 (5)	168

Symmetry code: (i) −*x*+*y*, −*x*+1, *z*−1/3.
